# Comparative performance of ChatGPT-5 and DeepSeek on the Chinese ultrasound medicine senior professional title examination

**DOI:** 10.3389/fdgth.2026.1783347

**Published:** 2026-03-09

**Authors:** Dao-Rong Hong, Chun-Yan Huang, Jiu Gao

**Affiliations:** 1 Department of Ultrasonography, The Second Affiliated Hospital of Fujian Medical University, Quanzhou, Fujian, China; 2 Department of General Practice, The Second Affiliated Hospital of Fujian Medical University, Quanzhou, Fujian, China

**Keywords:** artificial intelligence, ChatGPT-5, DeepSeek, medical education, professional title examination, ultrasound medicine

## Abstract

**Background:**

Large language models (LLMs) have shown growing potential for medical education and assessment, but evidence on their performance in specialty certification exams in China—particularly in ultrasound medicine—remains limited.

**Objective:**

To compare the performance of ChatGPT-5 and DeepSeek on the Chinese Ultrasound Medicine Senior Professional Title Examination, overall and by item type.

**Methods:**

Between August and September 2025, we randomly selected 100 multiple-choice questions from the official Chinese Ultrasound Medicine Senior Professional Title Examination bank (60 image-based interpretation items and 40 text-based items). We evaluated ChatGPT-5 and DeepSeek using identical prompts through their public web interfaces. The primary outcome was overall accuracy; secondary outcomes were accuracy by item type and subspecialty. Between-model differences were assessed using two-proportion *z*-tests (*α* = 0.05) in Python 3.12.

**Results:**

Overall accuracy was higher for ChatGPT-5 than for DeepSeek [74.0% (74/100) vs. 60.0% (60/100); *p* = 0.035]. Accuracy on image-based items was also higher for ChatGPT-5 (61.7% vs. 40.0%; *p* = 0.018). Performance on text-based items was similar for both models (92.5% vs. 90.0%). Subspecialty patterns varied across domains; however, no between-model differences reached statistical significance.

**Conclusions:**

ChatGPT-5 outperformed DeepSeek on image-based items (61.7% vs. 40.0%), while both models performed similarly on text-based knowledge items (92.5% vs. 90.0%). Overall, both LLMs showed strong performance on Chinese ultrasound senior-title examination questions, with complementary strengths across content areas. They may be useful as supplementary educational tools, but further advances in multimodal reasoning are needed to support more reliable image interpretation.

## Introduction

Artificial intelligence (AI) is increasingly embedded in modern health care, with demonstrated utility in clinically oriented tasks including imaging-based risk prediction and diagnostic support (1–3). For example, deep learning has been applied to develop malignancy potential prediction models in radiology-adjacent settings, illustrating its capacity to extract clinically relevant patterns from complex data (4). AI has also been discussed as an enabling technology for point-of-care testing (POCT), supporting timely decision-making and operational efficiency in near-patient diagnostic pathways (5).

In parallel, large language models (LLMs) have attracted substantial attention for their ability to generate medically coherent text and answer exam-style questions, positioning them as potential tools for medical education and assessment (6). Systematic evidence syntheses have summarized emerging applications and limitations of ChatGPT in specialty contexts, highlighting both promise and unresolved concerns regarding reliability and context dependence (7).

A common approach to benchmarking LLMs is to evaluate performance on standardized medical examinations and multiple-choice question (MCQ) banks, which enables controlled head-to-head comparisons when the question set, prompts, and scoring rules are prespecified (8–10). However, MCQ-based evaluations may overestimate clinical capability, because the stem and options provide bounded information and do not fully capture real-world information gathering and application. Recent analyses have highlighted an “evaluation illusion” of MCQ-dominant benchmarks and reported that models scoring highly on MCQs can still underperform on clinical-reasoning assessments such as script concordance testing. Notably, many exam-style LLM evaluations remain predominantly text-only and omit visual inputs, whereas multimodal benchmarks integrating medical images with exam-style questions are comparatively less common. In this context, the Chinese Ultrasound Medicine Advanced Professional Title Examination provides a distinct multimodal paradigm combining image-based ultrasound interpretation with text-based knowledge items, allowing joint assessment of visual and language-based reasoning.

Therefore, this study directly compares two contemporary LLMs (ChatGPT-5 and DeepSeek) using the Chinese Ultrasound Medicine Advanced Professional Title Examination question bank, stratified by item modality (image-based vs. text-based) and subspecialty. By quantifying overall accuracy and modality-specific performance, we aim to identify where current LLMs are most reliable for exam preparation and where limitations in multimodal reasoning persist, providing evidence relevant to ultrasound education and AI-assisted training strategies.

## Methods

### Question sampling and composition

We selected 100 multiple-choice items from the official bank maintained by the Ultrasound Medicine Branch of the Chinese Medical Association using a stratified random sampling design: 20 items were randomly sampled without replacement from each of five subspecialty domains (abdominal, OB/GYN, vascular, cardiac, superficial organ ultrasound). Randomization applied to item selection; the content of each item (stem, options, and images) was preserved as originally provided. Regarding accessibility, the bank is maintained as an internal assessment resource, and direct access is generally restricted to authorized users (e.g., examinees/authorized training programs/committee use, depending on local implementation). However, in practice, subsets of senior-title examination questions (or closely similar items) may be circulated via commercial test-preparation websites/apps, so inclusion of overlapping content in LLM training corpora cannot be fully excluded. The set included 60 image-based questions (sonographic image interpretation) and 40 text-based questions, covering abdominal, obstetric/gynecologic (OB/GYN), vascular, cardiac, and superficial organ ultrasound. Subspecialty domains and weighting. Items were sampled from five major domains (abdominal, OB/GYN, vascular, cardiac, superficial organ ultrasound) as tagged in the question bank; ambiguous tags were adjudicated by two ultrasound-physician authors. This fixed per-domain allocation was prespecified to enable balanced subspecialty-level comparisons and does not represent the official exam blueprint. The official subspecialty blueprint/weighting of the full examination is not publicly available (or not accessible to the authors); thus our stratified sample is not intended to estimate the exam-wide distribution. We did not use third-party “memory” prompts or retrieval tools, and the evaluation was performed offline without web browsing. We also included a large proportion of image-based items with original sonographic images, which are less likely to appear verbatim in text-only web corpora. Nevertheless, because training datasets for closed-source LLMs are not fully disclosed, contamination cannot be definitively ruled out.

### Evaluation protocol

#### Model access and session controls

Both ChatGPT-5 and DeepSeek were accessed via their official public web interfaces during August–September 2025 using dedicated logged-in user accounts created for this study. All evaluations were conducted in a private/incognito browser context. Before starting testing for each platform, we cleared cookies and site data for that platform and opened a fresh private/incognito window. Each exam item was then evaluated in a new chat/session with no follow-up turns, and responses were recorded verbatim.

#### Generation settings

We used each platform's default generation configuration available in the public web interface and did not tune decoding parameters. If any optional decoding controls were available (e.g., temperature/top-p), they were kept at default values and held constant across all items.

Responses were recorded in a pre-specified extraction sheet capturing question ID, item type, subspecialty, model response, selected option, and correctness. Before testing, both models received the same standardized instruction framing the model as an ultrasound specialist preparing for the senior-title examination. Standardized instruction (verbatim, Chinese): “你是一名超声医学专家, 正在准备超声医学高级职称考试. 请根据题干从 A–E 中选择一个最佳答案. 仅输出选项字母 (A/B/C/D/E)”. No few-shot examples were provided. We did not request step-by-step reasoning; outcomes were scored solely based on the selected option label. The full input template is provided in Supplementary Appendix 1. Items were presented one at a time using the original exam item format (stem + A–E options, and images when applicable). The order of presentation followed the study's randomized item list, rather than the original order of any complete examination form. For image-based items, sonographic images were provided in their original quality and layout.

#### Item format and presentation

Text-based items were entered as the original Chinese stem followed by five answer choices labeled A–E (single-best-answer format), pasted as plain text. Image-based items used the same A–E answer structure; the accompanying sonographic image(s) were uploaded directly in the chat interface without additional annotations or highlighting, preserving the original layout. If an item contained multiple images, they were uploaded in the original order as separate images within the same prompt. The model input for each item followed a fixed template (standardized role instruction + stem + options + “Answer with a single letter”), without additional reasoning prompts (e.g., chain-of-thought instructions). A representative input template and example items are provided in Supplementary Appendix 1.

#### Prompt language

All inputs (the standardized instruction and the question stems/options) were provided in Chinese, consistent with the original examination format. No translation step was used. For each item, we pasted the original Chinese stem and answer choices (A–E) verbatim; for image-based items, the original sonographic image(s) were uploaded alongside the Chinese text. We instructed models to respond by selecting a single best option (e.g., “A”); when provided, any explanatory text was also recorded verbatim. Outputs were accepted in Chinese, and correctness was scored based on the selected option.

Each item was evaluated in a separate new session for each model with no follow-up prompts.

#### Memory/personalization handling

For ChatGPT, the “Memory” feature and “Custom Instructions” were turned off in the account settings prior to data collection, and no additional personalization settings were used. For DeepSeek, we did not configure or apply any custom instruction profiles or personalization settings beyond the default web-interface configuration, and no memory/history-based features were intentionally enabled. These controls (logged-in status, private/incognito browsing, and cookie/site-data clearing) were implemented identically for both platforms to minimize cross-session carry-over and personalization effects. Because web-delivered systems may receive updates over time, our evaluation should be interpreted as a snapshot of the system available during August–September 2025.

### Outcomes

The primary endpoint was overall accuracy (percentage correct). Secondary endpoints were accuracy by item type (image- vs. text-based), subspecialty-specific performance, and inter-model agreement.

### Statistical analysis

We report accuracy as proportions (n/N). Between-model effect sizes were summarized as (i) absolute differences in accuracy (percentage-point difference) and (ii) relative differences (relative improvement of ChatGPT-5 vs. DeepSeek), with 95% confidence intervals where applicable. Between-model differences were assessed using two-proportion *z*-tests (two-sided, *α* = 0.05). Analyses were conducted in Python 3.12 using SciPy (stats) and statsmodels.

## Results

### Overall performance

**Figure 1:**
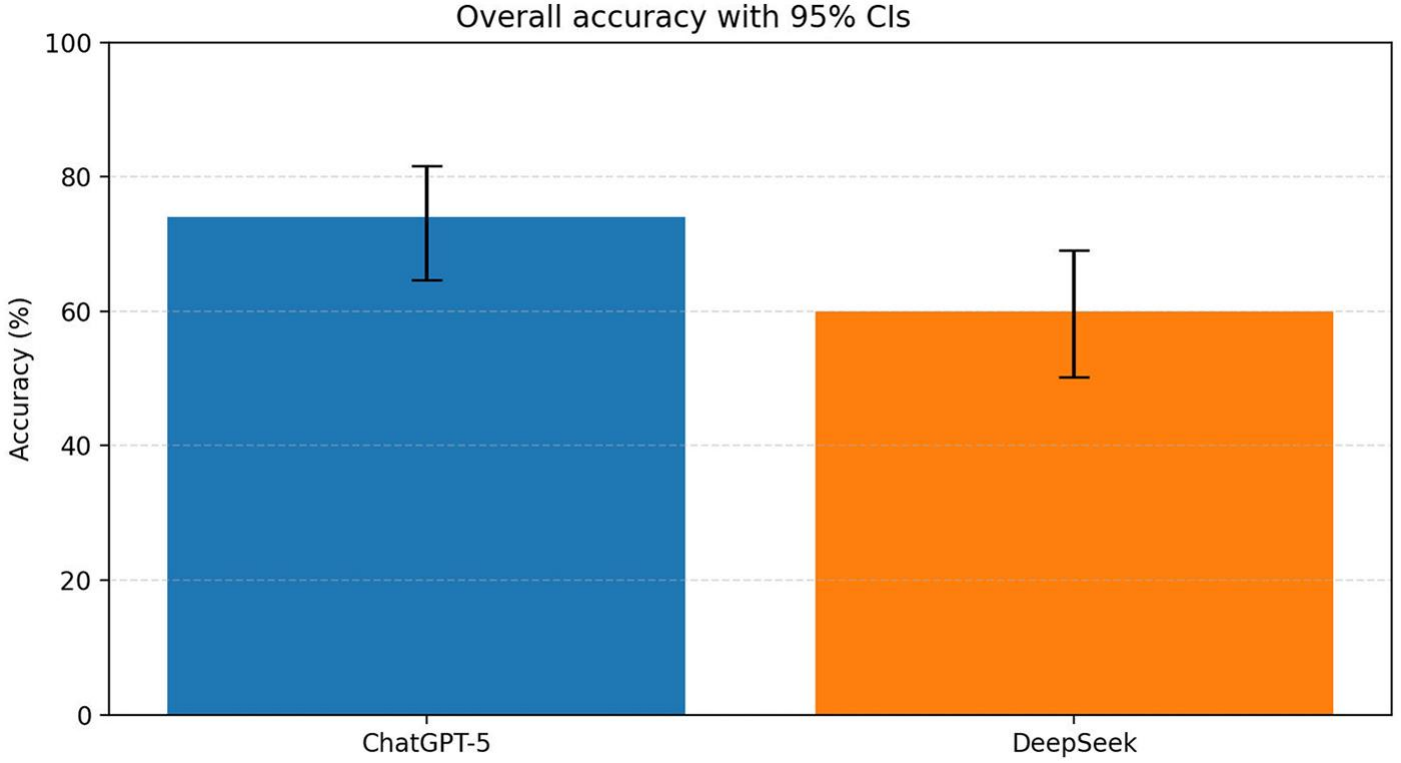
Overall accuracy of ChatGPT-5 and DeepSeek with 95% CIs. Comparison of overall accuracy between ChatGPT-5 and DeepSeek on the Chinese Ultrasound Medicine Senior Professional Title Examination. Error bars represent 95% confidence intervals. ChatGPT-5 demonstrated significantly higher overall accuracy compared to DeepSeek.

ChatGPT-5 achieved 74.0% accuracy (74/100), whereas DeepSeek achieved 60.0% accuracy (60/100), for an absolute difference of 14.0 percentage points (95% CI: 1.1–26.9) and a relative improvement of 23% [risk ratio (RR) of correct responses = 1.23; 95% CI: 1.01–1.50]. This difference was statistically significant (*p* = 0.035; Figure 1; Table 1).

**Table 1:** Summary of overall and item-type performance.

Category	ChatGPT-5 accuracy	DeepSeek accuracy	*n*	*p*-value
Overall	74.0% (74/100)	60.0% (60/100)	100	0.035
Image-based	61.7% (37/60)	40.0% (24/60)	60	0.018
Text-based	92.5% (37/40)	90.0% (36/40)	40	0.648

### Item-Type performance

**Figure 2:**
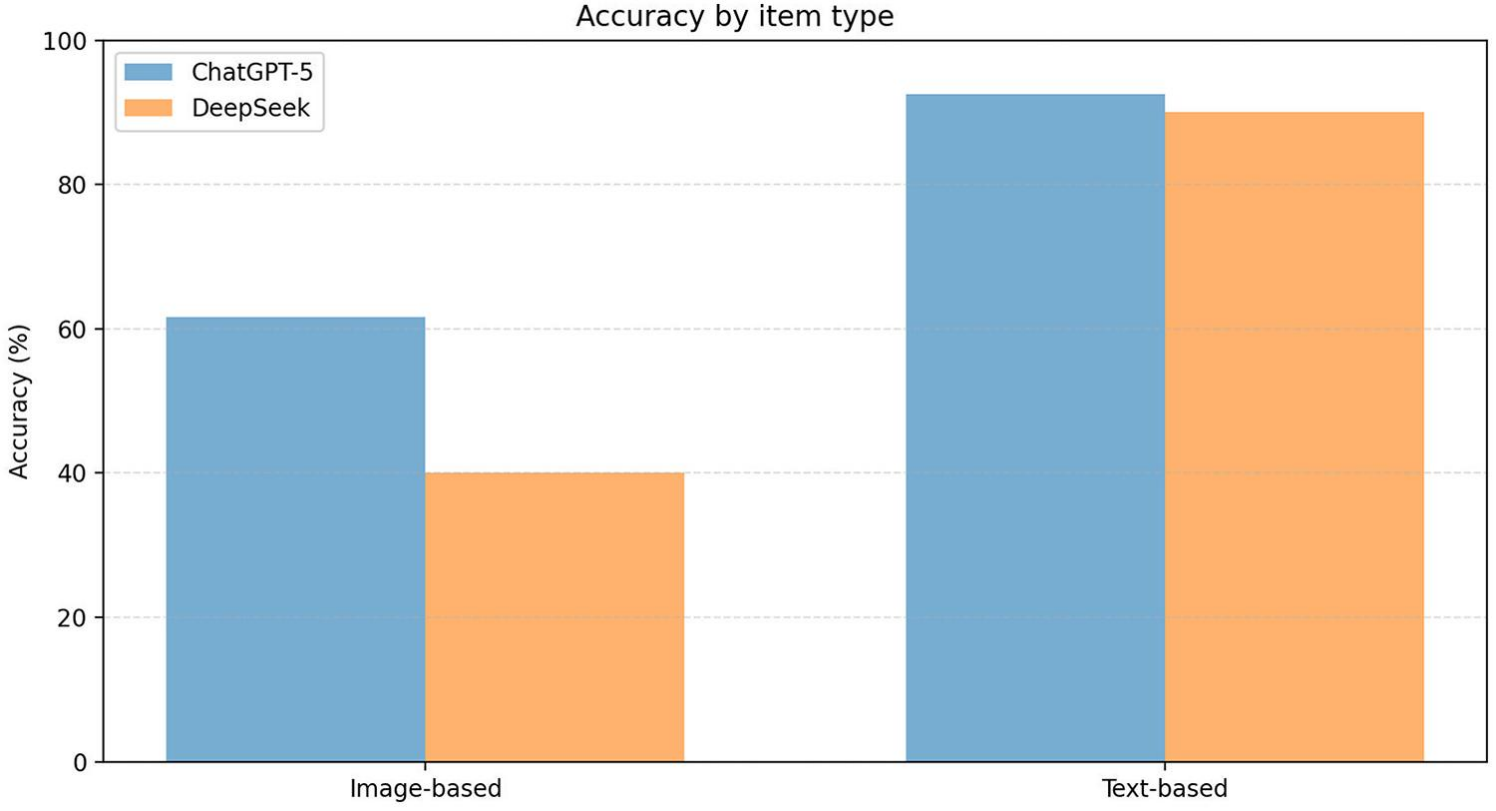
Accuracy by item type (image- based vs. text-based). Accuracy analysis by item type, comparing performance on image-based vs. text-based questions. Both models showed differential performance patterns across question types, with ChatGPT-5 showing higher accuracy on image-based items and similar near-ceiling accuracy on text-based items.

Performance differed by item modality (Figure 2; Table 1). For image-based items (*n* = 60), ChatGPT-5 outperformed DeepSeek [61.7% (37/60) vs. 40.0% (24/60)], an absolute difference of 21.7 percentage points (95% CI: 4.2–39.1; *p* = 0.018). For text-based items (*n* = 40), accuracy was near ceiling for both models [92.5% (37/40) vs. 90.0% (36/40)] with no statistically significant difference (*p* = 0.648).

### Subspecialty patterns

**Figure 3:**
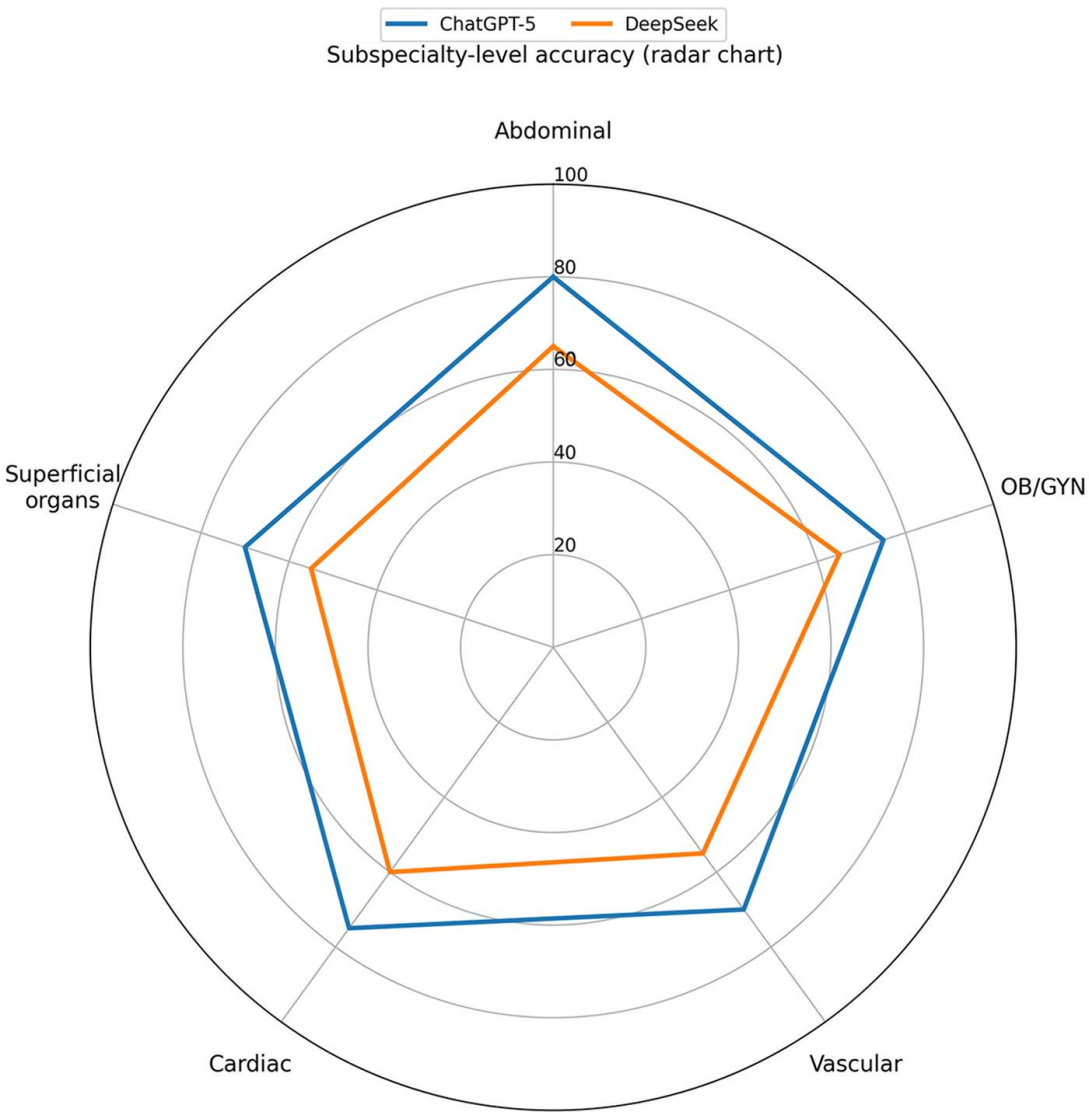
Subspecialty-level accuracy radar chart. Radar chart displaying subspecialty-level accuracy across five major ultrasound domains: Abdominal, Cardiac, Vascular, Superficial organs, and Obstetrics/Gynecology (OB/GYN). The chart reveals variable performance patterns between ChatGPT-5 and DeepSeek across different ultrasound subspecialties.

Across subspecialties, accuracy ranged from 70.0% to 80.0% for ChatGPT-5 and from 55.0% to 65.0% for DeepSeek (Table 2; Figure 3). None of the between-model comparisons reached statistical significance (*p* = 0.288–0.490). Absolute differences ranged from 10 to 15 percentage points. Each subspecialty contained 20 items (*n* = 20 per domain). This equal per-domain allocation was prespecified for analytic balance and does not represent official exam weighting.

**Table 2:** Accuracy by subspecialty and item modality (ChatGPT-5 vs. DeepSeek).

Subspecialty	Modality	*N*	ChatGPT-5 Accuracy	DeepSeek accuracy	*p*-value
Abdominal	Overall	20	80.0% (16/20)	65.0% (13/20)	0.288
	Image-based	12	75.0% (9/12)	50.0% (6/12)	0.227
	Text-based	8	87.5% (7/8)	87.5% (7/8)	1.000
OB/GYN	Overall	20	75.0% (15/20)	65.0% (13/20)	0.490
	Image-based	12	58.3% (7/12)	41.7% (5/12)	0.423
	Text-based	8	100% (8/8)	100% (8/8)	1.000
Vascular	Overall	20	70.0% (14/20)	55.0% (11/20)	0.327
	Image-based	12	66.7% (8/12)	33.3% (4/12)	0.227
	Text-based	8	75% (6/8)	87.5% (7/8)	0.500
Cardiac	Overall	20	75.0% (15/20)	60.0% (12/20)	0.311
	Image-based	12	58.3% (7/12)	41.7% (5/12)	0.423
	Text-based	8	100% (8/8)	87.5% (7/8)	0.500
Superficial organs	Overall	20	70.0% (14/20)	55.0% (11/20)	0.327
	Image-based	12	50.0% (6/12)	33.3% (4/12)	0.423
	Text-based	8	100% (8/8)	87.5% (7/8)	0.500

*p*-values were calculated using a two-sided two-proportion *z*-test (*α* = 0.05). Within each subspecialty, n_image + n_text = 20.

### Inter-model agreement

Exact-match agreement was 76.0% (76/100) and Cohen's kappa was 0.70 (95% CI: 0.60–0.80). Among disagreement items (*n* = 24), only ChatGPT-5 was correct in 19 (79.2%), whereas only DeepSeek was correct in 5 (20.8%), indicating that the overall performance gap was largely driven by items where the models diverged.

Overall, the statistically significant advantage for ChatGPT-5 was primarily driven by image-based questions (Figure 2; Table 1), whereas both models performed similarly on text-based questions.

## Discussion

### Summary of main findings

In this head-to-head evaluation of ChatGPT-5 and DeepSeek on the Chinese Ultrasound Medicine Senior Professional Title Examination, both models achieved substantial overall accuracy. ChatGPT-5 outperformed DeepSeek overall (74.0% vs. 60.0%), and this difference was mainly driven by image-based interpretation items (Table 1; Figures 1, 2). In contrast, both models achieved near-ceiling performance on text-based knowledge items (Table 1), suggesting that current LLMs may be more reliable for theory-oriented exam preparation than for ultrasound image interpretation. Importantly, ultrasound practice is inherently image-centered. The markedly lower accuracy on image-based interpretation items (61.7% for ChatGPT-5; 40.0% for DeepSeek) indicates that, in their current form, these systems should not be used for autonomous image interpretation or clinical decision-making. Instead, their present value is more defensible in educational and supportive roles (e.g., explaining concepts, guiding review of key imaging features), with qualified human oversight.

### Interpretation and comparison with prior studies

#### Text-based knowledge questions

The high accuracy we observed on text-based questions aligns with prior exam-style evaluations showing that LLMs perform well on knowledge-recall and guideline-driven multiple-choice items, particularly as model generations advance (8–10). In our dataset, the minimal between-model difference on text-based items suggests limited discriminative value when questions are primarily factual. More broadly, web-delivered LLM systems can vary across time and deployment settings; therefore, reproducible reporting should include the model identifier/label, access date, interface (web vs. API), and generation settings when user-adjustable (e.g., temperature/top-p).

#### Image-based interpretation questions

The higher accuracy of ChatGPT-5 on image-based items suggests better multimodal performance under our test conditions. However, this study was not designed to attribute the difference to a specific mechanism (e.g., vision encoder architecture, image preprocessing, alignment strategy, or decoding settings). Therefore, we interpret the result as a performance difference rather than evidence that one model has superior “feature extraction” *per se*. Conversely, the strong performance of both models on text-based items indicates that LLMs remain highly capable of handling theoretical knowledge questions, aligning with existing evidence showing LLMs' proficiency in medical knowledge tasks (8, 9, 11).

### Subspecialty patterns and inter-model disagreement

We observed exploratory subspecialty differences (Table 2; Figure 3), but none reached statistical significance, and the small per-domain sample size (*n* = 20) limits inference. Therefore, any apparent subspecialty advantage should be interpreted cautiously. Rather than reflecting true domain expertise, these patterns may arise from heterogeneous exposure to question styles and terminology in training data, as well as random variation in a limited item set. Notably, overall differences were concentrated in items where the models disagreed (Table 3), suggesting that performance gaps may be driven by a subset of clinically nuanced or visually complex questions. For educational use, this supports a complementary workflow in which learners compare model outputs, flag discordant cases for focused review, and rely on expert supervision for image-centric decisions.

**Table 3:** Inter-model agreement (option-label level).

Agreement type	Items (*n*)	Percentage (%)
Full agreement	76	76.0
Both correct	55	72.4^a^
Both incorrect (same option)	21	27.6^a^
Disagreement	24	24.0
Only ChatGPT-5 correct	19	79.2^b^
Only DeepSeek correct	5	20.8^b^
Both incorrect (different option)	0	0.0^b^
Cohen's kappa (κ)	–	0.70 (95% CI: 0.60–0.80)

^a^ Percentage within Full agreement items (*n* = 76).

^b^ Percentage within Disagreement items (*n* = 24).

Agreement was defined as selection of the identical option label (A–E) for a given item (free-text explanations were not used to define agreement).

### Comparison with prior studies

To place these results in context, we compare our findings with prior evaluations of LLMs on medical examinations and image-rich assessments, focusing on two recurring patterns: strong text-only performance and weaker image interpretation.

Our findings are consistent with the broader body of literature on the use of AI models in medical education. Previous studies have shown that AI models like ChatGPT can achieve high accuracy in medical exams, particularly in text-based tasks such as diagnosis and knowledge recall (10, 12–17). The most pronounced finding in our study is the significant performance gap between text-based items and image-based interpretation items. This discrepancy underscores that image understanding remains a critical bottleneck for current LLMs (18–20), a challenge consistently documented across medical specialties. Our results are strongly corroborated by studies evaluating LLMs on image-rich examinations. Al-Naser et al. reported that ChatGPT-4's accuracy dropped from 86.3% on text-based questions to 45.6% on image-based questions in the American Registry of Radiologic Technologists exam (20). Similarly, Maruyama et al. found that while GPT-4o significantly outperformed GPT-4 on text-only questions from the Japan Surgical Board Examination (78% vs. 55%), the inclusion of images did not lead to a significant improvement in performance, and accuracy on image-based questions remained substantially lower (21). Even models specifically evaluated on image identification tasks, such as Google Bard in an Objective Structured Practical Examination (OSPE) format, achieved an overall accuracy of only 56.7% (22). This collective evidence suggests that while LLMs excel at textual knowledge retrieval, their ability to interpret complex medical images—a cornerstone of diagnostic specialties like ultrasound medicine—is still developing and highly dependent on the quality of image annotation or integrated vision capabilities (19, 21).

#### Clinical relevance

The performance gap between text-only and image-based items has direct implications for ultrasound practice, where diagnostic accuracy depends on recognizing subtle image patterns (e.g., echogenicity, lesion borders, posterior acoustic features, and Doppler flow) in clinical context. Errors in image interpretation may translate into missed or incorrect diagnoses and inappropriate follow-up decisions. Our findings align with prior exam-based evaluations showing that LLM accuracy often drops substantially when images are required, reinforcing that visual reasoning remains a major limitation for current general-purpose LLM systems (17, 19–22).

Why did text-only items outperform image-based items? Text-only MCQs may benefit from broader representation in large-scale text corpora and may also be more susceptible to data contamination if similar questions circulate in preparation materials. In contrast, ultrasound image interpretation likely requires richer and more diverse visual training data (images plus reliable labels) than is currently available to general-purpose models. Although performance on image-based items was above chance, the observed error rates indicate that current visual knowledge remains insufficient for reliable primary interpretation.

Taken together, these findings suggest that apparent between-model differences may be driven largely by multimodal items, and our study extends this evidence to a Chinese specialty senior-title examination that jointly tests ultrasound image interpretation and text-based knowledge.

### Implications of the study

Our results suggest that current LLMs are better suited for theory-oriented learning and formative assessment than for primary ultrasound image interpretation. Given the substantially lower accuracy on image-based items, any deployment in ultrasound should be limited to supervised, assistive use cases (e.g., generating study explanations, prompting learners to check key sonographic features, or drafting differential considerations after a human interpreter has identified salient findings), rather than unsupervised diagnostic use. The better performance of ChatGPT-5 on image-based items suggests that, with further development, LLMs may contribute more meaningfully to image interpretation. LLM-assisted training should therefore prioritize improving multimodal reasoning, enhancing explanation quality, and reducing uncertainty in image-based outputs. Human oversight remains essential—especially for complex or ambiguous images—to avoid overreliance on AI-generated content.

In practice, LLMs may be best suited for formative applications, such as generating explanations, highlighting key diagnostic features, and supporting self-testing with immediate feedback. For image-based learning, outputs should be paired with structured checklists (e.g., probe position, key landmarks, differential diagnosis) and reviewed by supervisors to reduce the risk of plausible but incorrect interpretations.

### Limitations of the study

Platform comparability. Our comparison is between two consumer web deployments rather than isolated base models. Platforms may differ in weights (open-weight vs. proprietary), multimodal pipelines, and user-adjustable decoding settings. Because we tested default web configurations without tuning, results should be interpreted as a snapshot of these deployments rather than evidence of mechanistic superiority.

Implementation considerations in resource-constrained settings. Real-world deployment will depend on practical constraints (hardware/connectivity, cost, localization, data governance, regulation, workflow integration, and training). LMIC implementation studies emphasize that adoption and sustainability are context-dependent (23), and recent commentaries call for prospective, deployment-focused evaluations (24). Accordingly, use should be framed as supervised, assistive in resource-constrained settings. China is classified by the World Bank as an upper-middle-income economy; however, many ultrasound training and service settings remain resource-constrained, and LMIC-relevant implementation barriers remain applicable.

Because portions of professional title examination questions may be publicly circulated, potential overlap with LLM training data (data contamination) cannot be excluded, which may inflate performance estimates. The sample of 100 items from a single examination system may limit generalizability. In addition, we did not compare performance with human examinees (e.g., residents or fellows), and reliance on public web interfaces may introduce variability. Future studies should use larger, more diverse datasets, repeated version-tracked evaluations, and human comparators.

### Future directions

Future evaluations should incorporate repeated testing across model updates with explicit version tracking, pre-specified prompts, and (where feasible) human comparators to clarify when LLM assistance adds educational value and when it may mislead. Standardized reporting of model identity, query date, and evaluation workflow would improve reproducibility and comparability across studies.

## Conclusions

ChatGPT-5 outperformed DeepSeek on image-based ultrasound interpretation, while both models performed similarly on text-based knowledge items. These findings suggest that LLMs can support ultrasound education, with complementary strengths across content areas. Continued advances in multimodal reasoning are needed to improve image interpretation, and human oversight remains essential for complex image-based tasks.

## Data Availability

The original contributions presented in the study are included in the article/Supplementary Material, further inquiries can be directed to the corresponding authors.
