# Supplementary material for: Comparative performance of ChatGPT-5 and DeepSeek on the Chinese ultrasound medicine senior professional title examination
Source: Front Digit Health. 2026 Mar 9;8:1783347. doi: 10.3389/fdgth.2026.1783347 (PMC12968994; doi:10.3389/fdgth.2026.1783347)
Supplement: Supplementary file 1 [file Supplementaryfile1.docx]

Appendix 1

A. Fixed input template (used for all items)

1. Standardized role instruction (English translation of the Chinese): “You are an ultrasound medicine specialist preparing for a senior professional title examination in ultrasound medicine. Based on the item stem, select the single best answer from A–E. Output only the option letter.”

2. Item stem: pasted in Chinese (verbatim in the study; not reproduced here due to restrictions).

3. Answer choices: five options labeled A–E (single-best-answer).

4. For image-based items: associated sonographic image(s) were uploaded in the original layout/order without additional annotations or highlighting.

Important note: The examples below are paraphrased exemplars created by the authors to illustrate the structure and complexity of the inputs; they do not reproduce copyrighted exam items verbatim.

⸻

B. Representative text-based example (paraphrased exemplar)

Paraphrased stem (complete, readable):

“A 52-year-old man was found on routine health screening to have a focal hepatic lesion in the right lobe. He has a 15-year history of chronic hepatitis B. Ultrasound shows a hypoechoic nodule in the right hepatic lobe with relatively well-defined margins and heterogeneous internal echotexture. Which of the following additional tests/management steps would be most helpful to clarify the nature of the lesion and guide subsequent management?”

Options (A–E, single-best-answer):

A. Repeat standard non-contrast abdominal ultrasound and follow up in 3 months

B. Perform contrast-enhanced ultrasound of the liver or dynamic contrast-enhanced MRI/CT to evaluate the enhancement pattern

C. Proceed directly to percutaneous biopsy immediately without further imaging assessment

D. Obtain an abdominal plain radiograph first to rule out calcification

E. Measure liver function tests alone to determine management

Example model input (formatted as used in the study; English translation of the Chinese input):

You are an ultrasound medicine specialist preparing for a senior professional title examination in ultrasound medicine. Based on the item stem, select the single best answer from A–E. Output only the option letter.

Stem: A 52-year-old man was found on routine health screening to have a focal hepatic lesion in the right lobe. He has a 15-year history of chronic hepatitis B. Ultrasound shows a hypoechoic nodule in the right hepatic lobe with relatively well-defined margins and heterogeneous internal echotexture. Which of the following additional tests/management steps would be most helpful to clarify the nature of the lesion and guide subsequent management?

Options:

A. Repeat standard non-contrast abdominal ultrasound and follow up in 3 months

B. Perform contrast-enhanced ultrasound of the liver or dynamic contrast-enhanced MRI/CT to evaluate the enhancement pattern

C. Proceed directly to percutaneous biopsy immediately without further imaging assessment

D. Obtain an abdominal plain radiograph first to rule out calcification

E. Measure liver function tests alone to determine management

Please output only one option letter.

⸻

C. Representative image-based example (paraphrased exemplar + textual image description)

Paraphrased stem (complete, readable):

“A 34-year-old woman, 7 weeks of amenorrhea, presents with scant vaginal bleeding. Pelvic ultrasound images (grayscale + color Doppler) are provided. Based on the imaging findings, what is the most likely diagnosis?”

Textual image description (what the model saw, described in words):

“Transvaginal two-dimensional grayscale ultrasound shows no definite intrauterine gestational sac. In the right adnexal region, there is a round mixed-echogenicity mass with relatively well-defined margins, containing an anechoic area surrounded by a relatively thick echogenic ring. Color Doppler demonstrates circumferential perilesional hypervascularity with a prominent ‘ring-of-fire’ pattern. A small amount of free fluid is present in the pelvis.”

Options (A–E, single-best-answer):

A. Early intrauterine pregnancy (normal gestational sac)

B. Incomplete miscarriage

C. Ectopic pregnancy (likely tubal pregnancy)

D. Ruptured ovarian corpus luteum cyst

E. Adenomyosis

Example model input (formatted as used in the study; English translation of the Chinese input):

You are an ultrasound medicine specialist preparing for a senior professional title examination in ultrasound medicine. Based on the item stem, select the single best answer from A–E. Output only the option letter.

(In the same conversation, image files were uploaded in the original order: Grayscale image 1, Color Doppler image 2; no additional annotations or highlighting were added.)

Stem: A 34-year-old woman, 7 weeks of amenorrhea, presents with scant vaginal bleeding. Pelvic ultrasound images (grayscale + color Doppler) are provided. Based on the imaging findings, what is the most likely diagnosis?

Options:

A. Early intrauterine pregnancy (normal gestational sac)

B. Incomplete miscarriage

C. Ectopic pregnancy (likely tubal pregnancy)

D. Ruptured ovarian corpus luteum cyst

E. Adenomyosis

Please output only one option letter.

Note on image sharing: If original sonographic images cannot be redistributed due to copyright/exam-security restrictions, we provide a textual description of key sonographic features (e.g., echogenicity, margins, posterior acoustic features, Doppler flow patterns, and ancillary findings) to communicate what the models were asked to process and how the input was structured.
